# Analyses of HIV-1 integrase sequences prior to South African national HIV-treatment program and availability of integrase inhibitors in Cape Town, South Africa

**DOI:** 10.1038/s41598-018-22914-5

**Published:** 2018-03-16

**Authors:** Dominik Brado, Adetayo Emmanuel Obasa, George Mondinde Ikomey, Ruben Cloete, Kamalendra Singh, Susan Engelbrecht, Ujjwal Neogi, Graeme Brendon Jacobs

**Affiliations:** 1 Division of Virology, Institute for Virology and Immunobiology, Faculty of Medicine, University of Wuerzburg, 97080 Wuerzburg, Germany; 2 Division of Medical Virology, Department of Pathology, Faculty of Medicine and Health Sciences, Stellenbosch University, Tygerberg, 7505 Cape Town, South Africa; 3 Division of Clinical Microbiology, Department of Laboratory Medicine, Karolinska Institute, University of Stockholm, Stockholm, Sweden; 4 CSCCD, Faculty of Medicine and Biomedical Sciences, University of Yaoundé, Yaoundé, Cameroon; 5 South African Medical Research Council Bioinformatics Unit, South African National Bioinformatics Institute, University of the Western Cape, Western Cape, South Africa; 6 Department of Molecular Microbiology and Immunology, Columbia, MO 65211 USA; 7 Christopher Bond Life Sciences Center, University of Missouri, Columbia, MO 65211 USA

## Abstract

HIV-Integrase (IN) has proven to be a viable target for highly specific HIV-1 therapy. We aimed to characterize the HIV-1 IN gene in a South African context and identify resistance-associated mutations (RAMs) against available first and second generation Integrase strand-transfer inhibitors (InSTIs). We performed genetic analyses on 91 treatment-naïve HIV-1 infected patients, as well as 314 treatment-naive South African HIV-1 IN-sequences, downloaded from Los Alamos HIV Sequence Database. Genotypic analyses revealed the absence of major RAMs in the cohort collected before the broad availability of combination antiretroviral therapy (cART) and INSTI in South Africa, however, occurred at a rate of 2.85% (9/314) in database derived sequences. RAMs were present at IN-positions 66, 92, 143, 147 and 148, all of which may confer resistance to Raltegravir (RAL) and Elvitegravir (EVG), but are unlikely to affect second-generation Dolutegravir (DTG), except mutations in the Q148 pathway. Furthermore, protein modeling showed, naturally occurring polymorphisms impact the stability of the intasome-complex and therefore may contribute to an overall potency against InSTIs. Our data suggest the prevalence of InSTI RAMs, against InSTIs, is low in South Africa, but natural polymorphisms and subtype-specific differences may influence the effect of individual treatment regimens.

## Introduction

Combination antiretroviral therapy (cART) has dramatically reduced HIV infection to a chronic and manageable disease resulting in a near-normal life expectancy^1,2^. However, cART has also led to the development of resistance-associated mutations (RAMs) and transmitted drug resistances (TDRs), which are associated with a higher rate of virological failure^3–5^. With rising levels of drug resistance and first-line cART failure, more patients will require second-line and salvage cART, which shows treatment efficacy in terms of viral suppression in 60% of the cases, but may be up to three times more expensive than the first-line cART^6,7^.

HIV-1 Integrase (IN) is responsible for the integration of the viral nucleic material into the host genomic DNA^8^. Integrase strand transfer inhibitors (INSTIs) are highly potent antiretroviral agents with durable efficacy, minimal toxicity and is internationally approved and used for both treatment-naïve and treatment-experienced patients^9–11^.

There are currently three US-Food and Drug Administration (FDA)-approved InSTIs: Raltegravir (RAL), Elvitegravir (EVG) and Dolutegravir (DTG). Two newer INSTIs, bictegravir (BIC) and cabotegravir (CAB), are presently under consideration^12^. The use of higher genetic barrier drugs such as Dolutegravir (DTG) is crucial to the success of salvage therapy to mitigate the emergence of resistant variants^13^. In 2007, the first INSTI RAL was approved for the treatment of patients infected with HIV-1, followed by EVG in 2012. These first-generation InSTIs are highly effective in the treatment of HIV-1-infected patients, but have a low barrier to resistance, resulting in the rapid emergence of RAMs^14,15^. DTG is a second-generation InSTI that was approved by the FDA in 2014^16^. It has a higher resistance barrier than that of RAL and EVG^17^. In the case of DTG, resistance is selected slowly *in vitro*, but has not emerged in studies of therapy-naïve patients until today^18,19^. When compared to an EFV based first-line regimen, patients receiving DTG have shown to be superior regarding viral suppression rates and had stabilized CD4^+^ T-Cell counts^20^. This is mainly attributed to better adherence and fewer discontinuation rates under treatment. The WHO, however, only names it as an alternative to the above-mentioned first-line regimen, as little research has been done on the use of DTG^21^.

Since its initiation in 2004, South Africa’s national HIV treatment program has grown to become the biggest in the world, currently treating approximately 3.4 million people^22^. Being in concordance with the World Health Organisations (WHO) guidelines, the recommended first-line combination antiretroviral therapy (cART) in South Africa consists of a non-nucleoside reverse transcriptase inhibitor (NNRTI) backboned regimen of Efavirenz (EFV), combined with two nucleoside reverse transcriptase inhibitors (NRTIs), namely Lamivudine (3TC) and either Tenofovir Disoproxil Fumarate (TDF) for adults or Abacavir (ABC) for children, respectively. The recommended second-line cART consists of the nucleoside reverse transcriptase inhibitors (NRTIs) Zidovudine (AZT) and Lamivudine (3TC) and a Ritonavir-boosted (/r) Protease Inhibitor (PI), usually Atazanavir^23^.

In this study we aim to provide further information on the susceptibility and primary drug resistance mutations profile of InSTIs as well as to establish a protocol to screen for Integrase RAMs in an HIV-1 subtype C predominated setting in South Africa.

## Results

### Patient demographics

The patient demographics are summarized in Supplementary Table 1. We amplified and confirmed successful sequencing, containing all 288 amino acids of the IN gene, for 91 samples.

### HIV-1 subtyping

**Figure 1:**
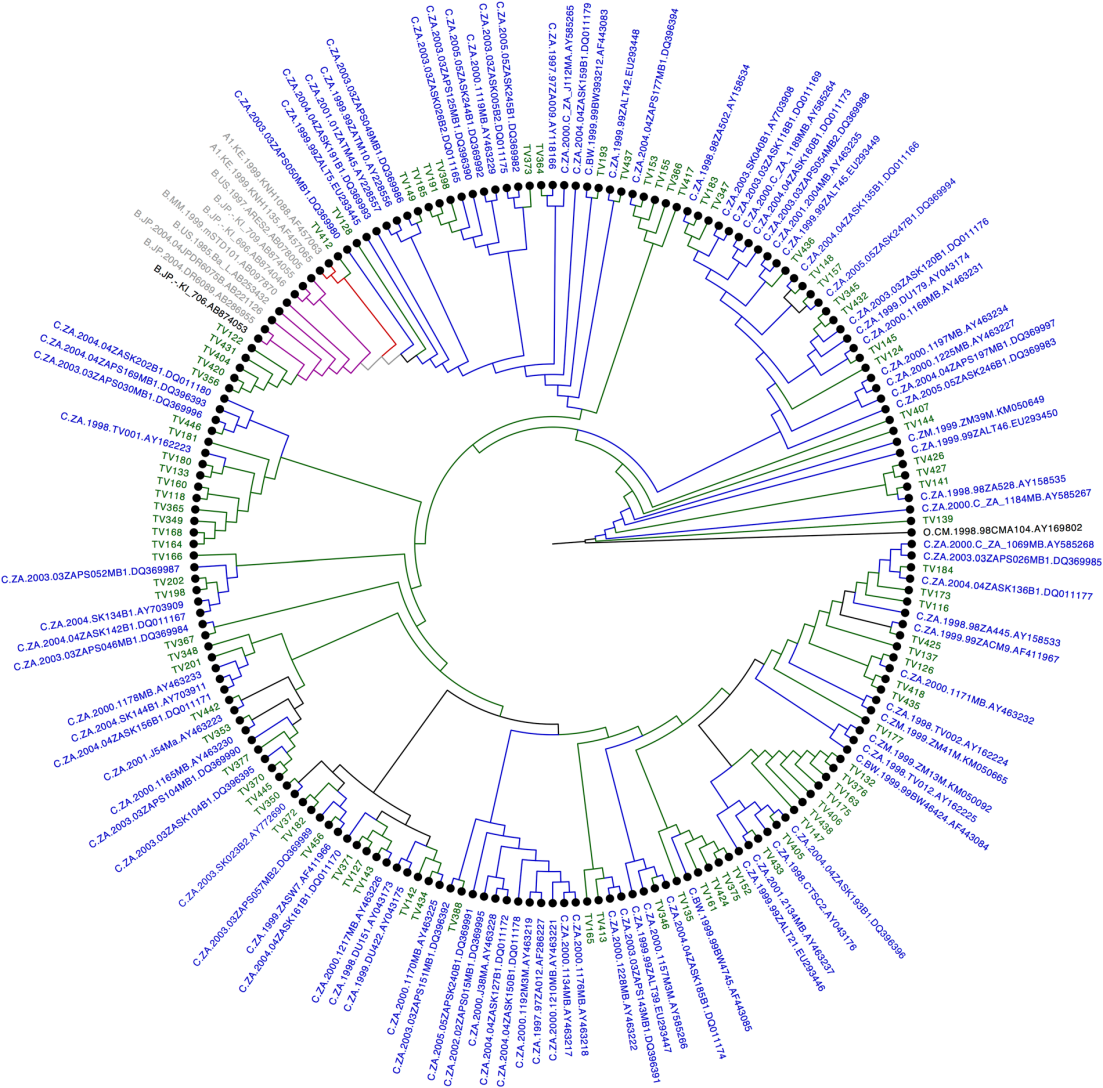
HIV-1 Integrase molecular phylogenetic analysis inferred by the maximum likelihood (ML) method. The ML phylogenetic tree inferred in RAxmL contains 91 patient sequences and HIV-1 reference sequences dataset from 2010 were acquired from HIV-1 LANL database. The evolutionary distances were computed using the general time reverse (GTR) model of nucleic acid substitution with an estimated Gamma shape parameter and invariant sites. The genetic distance is displayed in the scale bar at the bottom of the figure; while the majority of the sequences clusters with HIV-1 subtype C. 87 of the samples clustered with Subtype C reference strains (91.1%), five with Subtype B (5,6%) and one with Subtype A1 strains (1.1%).

Based on HIV-1 subtyping using online automated tools and phylogenetic analysis, 85 (92%) of the samples were identified as HIV-1 subtype C followed by five (5.4%) as HIV-1 subtype B (5,6%, TV122; TV431; TV404; TV420 and TV356) and one as HIV-1 subtype A1 strains (1.1%, TV412) (Fig. 1).

### Resistance mutation analyses

Drug resistance analyses showed that no major InSTI RAMs were present in this study. One sample (TV367) carried the accessory drug mutation G140E, a non-polymorphic mutation, that has been selected *in vitro* before, but which alone does not seem to influence the susceptibility of the virus to InSTIs^24^. Minor, polymorphic, mutations were present in 6/91 samples (6.6%), of which four samples contained the mutation L74I (TV122, TV128, TV173, TV405), one other sample contained the mutation L74M (TV366) and another the polymorphism S230N (TV364).

Of note is, that 55/91 (60.4%) samples carried the M50I polymorphism, all of which were classified as subtype C. M50I does not confer resistance to any of the currently available InSTIs and therefore is not listed as RAM in the Stanford University HIV Drug Resistance Database (https://hivdb.stanford.edu/). However, it has been selected *in vitro*, following a bictegravir (BIC) resistance selection assay^25^. In this M50I succeeded an R263K mutation, and only conferred low-level resistance to BIC (2.8-fold) in this combination. R263K was not present in our cohort.

### Database derived IN sequence resistance analyses

After excluding multiple sequences from a patient to avoid overestimation of the variant calling and problematic sequences, we used 314 sequences collected between 1999 and 2007. These, we subsequently screened for the presence of RAMs, and identified 6.4% (20/314) sequences to contain RAMs with only 2.86% (9/314) having major InSTI resistance mutations: Q148H, T66S, E92G, S147G, T66A, Y143YF as well as Y143H. Q148H, T66S, E92G, Y143YF, and S147G were present in one sequence each (0,3%) whereas T66A and Y143H could be detected in two sequences respectively (0.6%). 3,5% (11/314) of the sequences contained four different IN accessory mutations, namely E157Q, T97A, G163, and S230R.

### Generation of consensus South African HIV-1 subtype C sequence (HIV-1C_ZA_)

**Figure 2:**
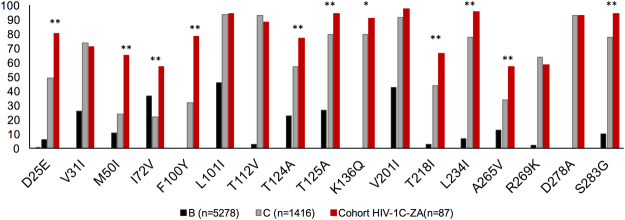
HIV-1C_ZA_ Integrase mutation profiling. Integrase mutation profiling of consensus sequences generated using the database-derived HIV-1C_ZA_ sequences HIV-1B (n = 5278), HIV-1C (n = 1416), cohort sequences HIV-1C-ZA (n = 87) identified 17 naturally occurring polymorphisms D25E, V31I, M50I, I72V, F100Y, L101I, T112V, T124A, T125A, K136Q, V201I, T218I, L234I, A265V, R269K, D278A and S283G. Among the 17 mutations 11 were further increased in our cohort

The consensus sequences generated using the database-derived HIV-1C_ZA_ sequences (n = 314) and cohort sequences (n = 85) identified 17 naturally occurring polymorphisms D25E, V31I, M50I, I72V, F100Y, L101I, T112V, T124A, T125A, K136Q, V201I, T218I, L234I, A265V, R269K, D278A, and S283G. (Fig. 2). Further profiling of sequences obtained through HIVseq Program, HIV-1B (n = 5278), HIV-1C (n = 1416), cohort sequences HIV-1C-ZA (n = 87) confirmed the findings. Among the 17 mutations 11 were further increased in our cohort.

### Molecular modeling

**Figure 3:**
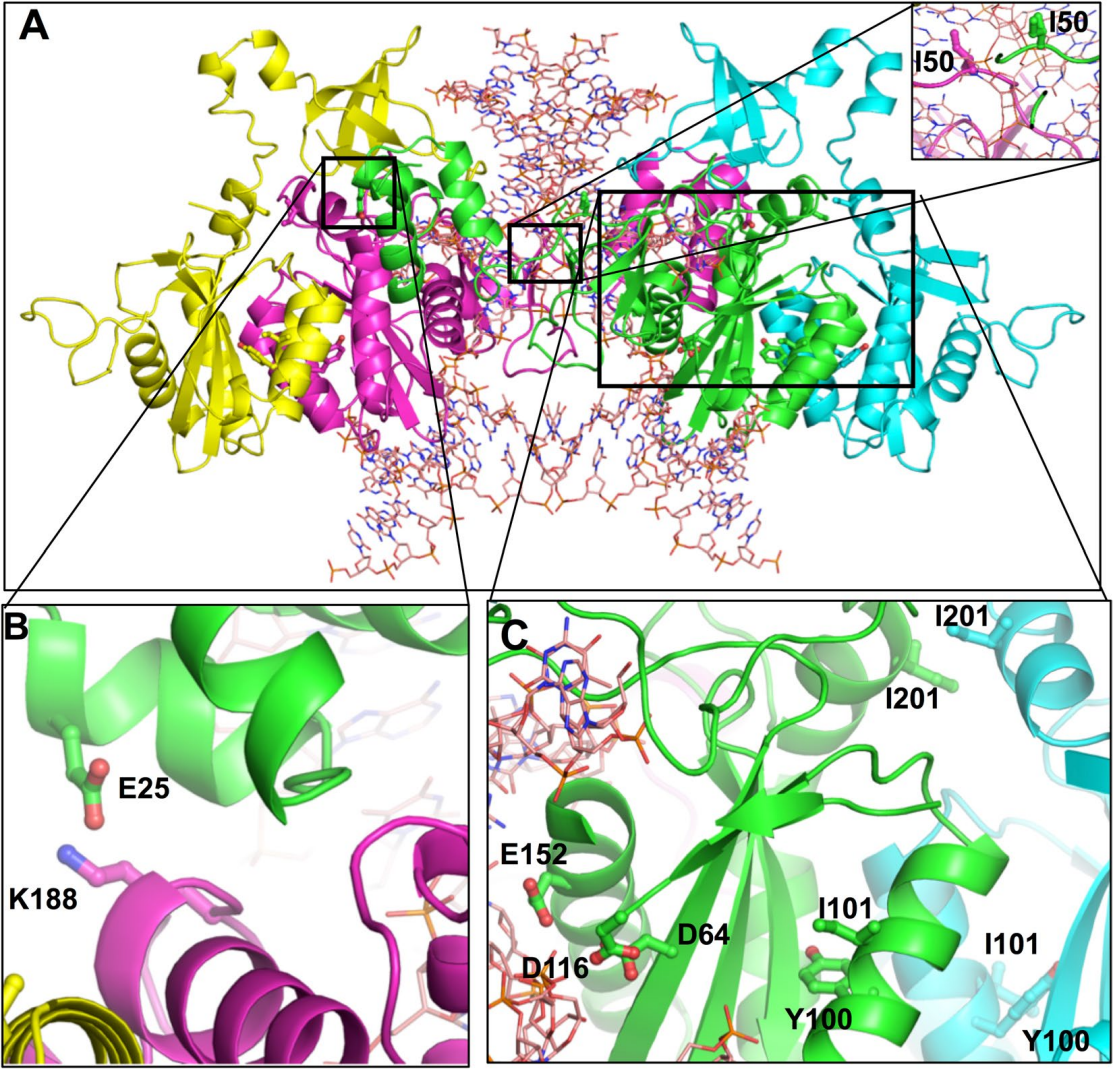
Homology derived molecular model of consensus HIV-1C_ZA._ Homology derived a molecular model of Con_C_ZA. Figure A shows an intasome consisting of a tetramer of subtype C_ZA and substrate DNA. This structure was generated using the cryoEM structure of HIV-1B IN intasome (PDB file 5U1C) using ‘Prime’ of Schrodinger Suit using the protocol discussed in Neogi *et al*., 2016. Inset in panel shows the proximity of I50 to DNA. Two I50 residues from two different subunit interact with DNA from two different sides. Figure B shows the position of E25 (in subunit colored green) that forms a ion-pair with K188 of subunit colored magenta. This is a symmetric interaction as E25 from magenta subunit interacts with K188 of green subunit. This interaction is important in maintaining the tetramer of IN. Figure C shows the active site residues D64, D116 and E152 of IN in one subunit (colored green) together with Y100 and I100 in the same and in the neighboring subunit. This figure also shows the position of I201 in two neighboring subunits. This interaction also appears critical for the maintenance tetramer organization of IN.

The molecular models were created using the cryoEM structure of HIV-1B IN (Figure 3). The cryoEM structure has an active site mutation E152Q, in the modeled structure, this mutation was reverted to glutamate. Here, we focussed on our five naturally occurring polymorphisms: E25, I50, Y100, I101 and I201 in the modeled structure of HIV-1C_ZA_ IN (Figure A). Our model showed that I50 (M50I mutation) is in the proximity of two strands of substrate DNA from two different monomers and therefore appears important in stabilizing/binding with DNA substrate (Figure B). Residue E25 (D25E mutation) from one monomer forms an ion-pair with K188 of a different monomer in a symmetric fashion (Figure C). The two monomers directly bind DNA substrate and have their active site positioned close to DNA. This interaction is essential in maintaining the tetramer of IN. Mutations Y100 (F100Y) and I101 (L101I) are close to the active site of IN (D64, D116, and E152) in two monomers of which one subunit binds DNA through the active site, and the other one has an indirect interaction. Mutation I201 (V201I) is juxtaposed at the interface of two monomers of IN proteomer forming a hydrophobic interaction suggesting its importance in the maintenance of IN/DNA complex.

## Discussion

InSTI containing regimens are considered a new and effective form of salvage therapy for cART-experienced patients failing first and/or second-line cART. South Africa, managing the most extensive HIV treatment program, has faced an increase in resistance rates against NNRTIs, NRTIs as well as PIs in the past years^26^. With second-generation InSTIs not being readily available until recently and drug resistance rates in cART naïve patients in some cases exceeding 10%, these drugs could play an essential role in maintaining treatment options against multi-drug-resistant virus variants and preventing resistant viruses from further spreading^5,27,28^

In this study we analyzed the IN region of HIV-1 infected, cART naïve patients, for the presence of InSTI treatment compromising polymorphisms and mutations. We showed that no primary major resistance mutations against InSTIs were circulating in our study cohort at the time of collection. One accessory mutation (G140E) was observed, while the highly polymorphic mutations L74I/M and S230N were present in 5,5% (5/91) and 1,1% (1/91), respectively. Neither of these mutations is associated with reduced susceptibility to InSTIs. L74I/M, a polymorphism, that has been described in both, cART naïve and RAL or EVG experienced patients before, does not diminish the effect of InSTIs by itself, but can contribute to a high-level resistance, only if co-occurring with major resistance mutations^29,30^. S230N has been reported as a natural variant with a polymorphism rate ranging between 0,5% to 2,0%^31^. It has also been selected by RAL and/or EVG before, *in-vivo* and *in-vitro*, but does not seem to confer resistance to any of the available drugs^31,32^.

These findings are in line with previous studies on cART naïve patients confirming the variability of the genomic IN region as well as its lack of major resistance mutations. In 2013 Bessong and Nwobegahay reported the absence of major RAMs in a study conducted in the north-eastern part of South Africa, and a year before, in 2012, Oliveira *et al*. analyzed HIV-1 positive samples from Mozambique for genetic diversity of the IN gene^33,34^. While resistance-associated mutations were not present in this study, the L74M polymorphism was found in 3,4% of the cases. Similar results were observed in Brazil and Europe, before the widespread use of InSTIs^35,36^.

Among the 9/314 (2.86%) major InSTI RAMs, present in the database-derived sequences, only Q148H, detected in 1/314 (0.3%), may profoundly affect second-generation InSTIs susceptibility. If co-occurring with additional RAMs, mutations in the Q148 pathway can lead to higher fold resistances against all InSTIs. Despite both first-generation InSTIs, RAL, as well as EVG, selecting for these mutations, they have not yet been described to emerge under initial second-generation InSTI treatment^37^. Y143H (2/314, 0.6%) is usually selected by RAL, and is considered to be a transitional mutation as part of the Y143R resistance pathway. Alone Y143H does not influence the effect of InSTIs, but by further mutating to Y143R it may confer moderate to high-level resistance to RAL, but minimal if any resistance to DTG^38,39^.

T66S, T66A, E92G and S147G, found in 0.3%, 0.6%, 0.3%, and 0.3%, respectively, are non-polymorphic mutations, normally selected by EVG treatment. They are associated with moderate to high-level resistance against EVG, although T66 mutations also bear cross-resistance to and are selected by RAL^40,41^^.^

The most frequent IN accessory RAM within the online, retrieved sequences was T97A, being present in 1.6% (5/314), followed by E157Q in 0.96% (3/314), G163R in 0.6% (2/314) and S230R in 0.3% (1/314) of the cases. All of these mutations are found to be within their natural prevalence rates, and although they can confer low-level resistance to both, RAL and EVG, none of these mutations are known to reduce DTG susceptibility, neither *in vitro* nor *in vivo*^41–44^.

Interestingly, one case report found single E157Q to be associated with treatment failure of a DTG containing regimen^45^. Therefore, Anstett *et al*. investigated this association in 2016, but could not confirm the result^46^. On the other hand, however, a recent study has also shown that eight patients, who had E157Q mutation and initiated with DTG-based therapy, did not suppress the viremia below detection level after six months of therapy^47^. Hence, causality between E157Q and a reduced DTG susceptibility is debatable and needs further long-term follow-up studies.

Despite higher fold RAMs against InSTIs being absent in most treatment naïve settings, they can emerge under treatment, particularly with first generation InSTIs, as Rossouw *et al*. presented in their case report from May 2016. In this report, they describe the first South African patient to fail EFV based first-line consecutively, ritonavir-boosted Lopinavir backboned second-line, and RAL containing third-line therapy^48^. Poor adherence to the therapy was reported throughout the patient’s history, and a final drug-resistance test, performed three years after the initiation of third-line treatment, for the first time included InSTI-resistance testing. This test ultimately confirmed a high-level resistance against RAL and high- or intermediate-level resistance against three of the other four drugs.

Furthermore, this test also revealed cross-resistance to EVG and a low-level resistance against DTG. This cross-resistance to DTG is seldom observed in the only RAL exposed patients and therefore is worrying, especially because InSTI resistances develop significantly less frequently if initially treated with DTG, instead of RAL^49,50^.

Nevertheless, this case study raises the concern of emerging InSTI resistance patterns in the South African context. Hence, proper drug resistance surveillance within South Africa will be required, in particular also because, a recent study identified subtype-specific differences in DTG cross-resistance patterns in patients failing RAL^39^. Further, sequence and structure-based analyses showed that the subtype-specific effects were caused by polymorphic residues across subtypes, which significantly affected native protein activity, structure and function of importance for drug-mediated inhibition of enzyme activity^51^. Although DTG showed a high genetic barrier to resistance, subtype-specific differences have been observed in the selection of DTG resistance mutations.

Therefore, we analyzed the position of naturally polymorphic mutations in the context of their ability to impact the stability of intasome. The polymorphisms noted in our analyses appear to be essential for the stability of tetramer and/or binding of DNA substrate in catalytically competent mode. The topological positions of polymorphisms also suggest that the intasome complex stability may differ in different subtypes, which may alter the architecture of the complex and thereby affect InSTI-based therapy outcome.

As the analysed cohort was recruited before the initiation of the HIV treatment program in South Africa, the possibility of RAMs being transmitted by treatment-experienced individuals is highly unlikely. Therefore, we consider the described findings to be a true baseline InSTI resistance rate.

Our data suggest that the introduction of this class of ART drugs, especially second-generation InSTIs, into the national treatment program could help in managing the HIV epidemic in South Africa. However, the possible emergence of formerly described, as well as a subtype and setting specific resistance pathways, requires proper drug resistance surveillance in the future, in order to track the evolution of the virus in a subtype C predominated setting under the pressure of the new treatment.

## Conclusion

In the absence of a cure for HIV, long-term cART outcomes need to be monitored efficiently for maximum efficiency. RAMs lead to therapy escape mutants, which can ultimately cause cART failure. We have shown that in the South African context InSTIs is potentially a viable option for salvage therapy. However, there is still a need to keep assessing the RAMs to ensure patients receive the best treatment and care possible.

## Methods

### Ethics statement

This study was approved by the Health Research Ethics Committee of Stellenbosch University, South Africa (N15/08/071). The study was conducted according to the ethical guidelines and principles of the international Declaration of Helsinki 2013, South African Guidelines for Good Clinical Practice and the Medical Research Council (MRC) Ethical Guidelines for Research. A waiver of consent was awarded to conduct analyses on the samples, as they were obtained between 2000 and 2001, stored since 2001.

### Study design

The samples used for analyses (n = 91) were part of a previously described Tygerberg Virology (TV) cohort^52^. The cohort contains treatment naïve patient samples from multiple ethnic groups, as well as different sexual orientations. Patients were sampled between 2000 and 2001, before the initiation of South Africa’s national HIV treatment program and the introduction of INSTIs.

### Collection of patient samples

Whole blood was collected from patients with ethylenediaminetetraacetic acid (EDTA) tubes. Plasma was separated after centrifugation at 2000 rpm for 10 minutes at 4 °C. The samples were stored at −80 °C for long-term storage. We selected randomly from the stored samples for IN amplification.

### Nucleic acid extraction

HIV-1 RNA extraction was performed using the QIAamp Viral RNA Mini Extraction Kit’s Spin protocol according to the manufacturer’s instructions (Qiagen, Germany). Briefly, 140 µl of plasma was used as a starting volume. Larger starting volumes of 280 µl of plasma was used for some samples with very low viral titers. Viral RNA was stored at −80°c until use.

### cDNA synthesis and PCR amplification

The synthesis of complementary DNA (cDNA) and first-round PCR amplification was performed using the Invitrogen SuperScript® III Reverse Transcriptase (RT) reagents (Invitrogen, Germany). HIV-1 Integrase specific primers used for amplification were Poli5 (5′- CACACAAAGGRATTGGAGGAAATG-3′) and Poli8 (5′-TAGTGGGATGTGTACTTCTGAAC-3′), position 4162–4185 and 5195–5217 on the HXB2 reference strain, respectively, with an expected fragment size of 1056 base pairs^53^. The 25 ${\rm{\mu }}{\rm{l}}$ reaction volume contained 8,5 ${\rm{\mu }}{\rm{l}}$ nuclease-free water 12,5 ${\rm{\mu }}{\rm{l}}$ of 2 × reaction mix buffer, 0.5 ${\rm{\mu }}{\rm{l}}$ of both forward and reverse primers at a concentration of 5nmol, 0.5 ${\rm{\mu }}{\rm{l}}$ of RT/Platinum Taq mix and 2,5 ${\rm{\mu }}{\rm{l}}$ of extracted RNA. Reverse transcription was performed at 50 °C for 20 minutes, followed by an initial denaturation at 94 °C for 2 minutes. Forty cycles of amplification were carried out at 94 °C for 15 seconds for denaturation, 30 seconds for primer annealing at 94 °C and 90 seconds for elongation at 68 °C. The final elongation step was done at 68 °C for 10 minutes. For second round PCR amplification, primers Poli7 (5′-AACAAGTAGATAAATTAGTCAGT-3′) and Poli6 (5′-ATACATATGRTGTTTTACTAARCT-3′), with an expected fragment size of 945 base pairs relative to position 4186–4209 and 5107–5130, were used, in combination with the GoTaq® Flexi DNA Polymerase Kit (Promega, USA)^53^. The 50 ${\rm{\mu }}{\rm{l}}$ reaction mix consisted of 28.5 ${\rm{\mu }}{\rm{l}}$ nuclease-free water, 10 ${\rm{\mu }}{\rm{l}}$ of 5 × reaction mix buffer, 0.5 ${\rm{\mu }}{\rm{l}}$ of both primers at 20 nmol, 3.0 ${\rm{\mu }}{\rm{l}}$ of MgCl_2_ at 75 nmol and 3 ${\rm{\mu }}{\rm{l}}$ of amplified DNA. Second round of amplification started with a denaturation at 95 °C for 2 min, followed by 40 cycles of denaturation at 95 °C for 20 seconds, annealing at 55 °C for 30 seconds and elongation at 72 °C for 90 seconds. The final elongation step was done at 72 °C for 10 minutes and amplicons were stored at 4 °C until further use. Positive PCR amplicons were purified from agarose gel according to the manufacturer instructions using the QIAquick PCR Purification Kit (Qiagen, Germany).

### Sanger DNA Sequencing

All amplicons were sequenced on both strands with conventional Sanger DNA sequencing using the ABI Prism Big Dye® Terminator sequencing kit version 3.1 and run on the ABI 3130xl automated DNA sequencer (Applied Biosystems, USA). The sequencing reactions were performed according to the manufacturer’s instructions. Briefly, sequencing PCR cycling conditions were as follows: Initial denaturation of 95 °C for 60 seconds, followed by 25 cycles of 95 °C for 60 seconds, 55 °C for 7 seconds and 60 °C for 4 minutes. Sequencing primers consist of the above Poli6 and Poli7. Additional sequencing primers were used namely; Poli2 (TAAARACARYAGTACWAATGGCA), relative to position 4745–4766 and KLVO83 (GAATACTGCCATTTGTACTGCTG), corresponding to position 4750–4772^54^. After that, sequences were assembled into contiguous fragments following (Phred quality score > 20) and edited manually using Sequencer version 5.0 (Gene Codes Corporation, USA). The bases were considered ambiguous is any nucleotide was present > 25% of the major peak.

### HIV-1 Subtyping and phylogenetic analyses with online programs

HIV-1 subtyping based on integrase sequences was carried out using REGA v3 and COMET-HIV, followed by maximum likelihood phylogenetic analysis^55^. The best fitted general time reverse (GTR) model of nucleic acid substitution with an estimated Gamma shape parameter and invariant sites model using Randomized Axelerated Maximum Likelihood (RAxML) as described previously^56,57^.

### Additional sequences

To compare our sequences with the rest of the IN sequences from South Africa, we performed a search on the LANL HIV database (https://www.hiv.lanl.gov/components/sequence/HIV/search/search.comp). Our search inclusion criteria included all South African IN sequences and those identified from treatment naïve patients. We selected one sequence per patient and all problematic sequences were excluded from further analyses. Finally, 314 HIV-1 subtype C (HIV-1C) sequences were included in the analyses. Both cohort and database derived South African IN sequences were used to generate the consensus HIV-1C_ZA_ sequence using the Consensus Maker tool available in HIV-1 Los Alamos database using majority value 0.5 (https://www.hiv.lanl.gov/content/sequence/CONSENSUS/consensus.html). HIVseq Program, a literature prevalence of mutations in submitted sequences were used to identify the prevalence of naturally occurring polymorphisms in the HIV-1C_ZA_ sequences in HIV-1B^58^.

### Molecular Modelling

The homology model of HIV-1C_ZA_ IN tetramer was generated using the cryoEM structure of HIV-1B IN intasome (PDB file 5U1C) in the presence of DNA substrate, using Prime version 4.2 of the Schrodinger Suite (Schrodinger, New York, NY, USA), integrated into Maestro of Schrodinger Suite, (Schrodinger Inc., NY) as described previously^59,60^^,^ The homology model was subjected to energy minimization (5,000 steps) to reduce steric overlap between residues using the “Impact” utility of the Schrödinger Suite and the OPLS_2005 force field as described before^61^. The modeled structure was submitted to the Structure Analysis and Verification Server (SAVES) (https://services.mbi.ucla.edu/SAVES/) as well as Protein Structure Preparation tool of SYBYL-X (version 2.1). No bad contacts were noted in the structures. The backbone torsion angles were checked by Ramachandran plot for allowed conformations of ϕ and φ angles. All angles were in the allowed range. The mutant modeling was conducted with ‘Prime’ utility of Schrodinger Suite Fig. 3.

## Electronic supplementary material

Supplementary Table 1
