# Author Correction: Analyses of HIV-1 integrase sequences prior to South African national HIV-treatment program and availability of integrase inhibitors in Cape Town, South Africa

**DOI:** 10.1038/s41598-018-24183-8

**Published:** 2018-04-16

**Authors:** Dominik Brado, Adetayo Emmanuel Obasa, George Mondinde Ikomey, Ruben Cloete, Kamalendra Singh, Susan Engelbrecht, Ujjwal Neogi, Graeme Brendon Jacobs

**Affiliations:** 1 Division of Virology, Institute for Virology and Immunobiology, Faculty of Medicine, University of Wuerzburg, 97080 Wuerzburg, Germany; 2 Division of Medical Virology, Department of Pathology, Faculty of Medicine and Health Sciences, Stellenbosch University, Tygerberg, 7505 Cape Town South Africa; 3 Division of Clinical Microbiology, Department of Laboratory Medicine, Karolinska Institute, University of Stockholm, Stockholm, Sweden; 4 CSCCD, Faculty of Medicine and Biomedical Sciences, University of Yaoundé, Yaoundé, Cameroon; 5 South African Medical Research Council Bioinformatics Unit, South African National Bioinformatics Institute, University of the Western Cape, Western Cape, South Africa; 6 Department of Molecular Microbiology and Immunology, Columbia, MO 65211 USA; 7 Christopher Bond Life Sciences Center, University of Missouri, Columbia, MO 65211 USA

Correction to: *Scientific Reports* 10.1038/s41598-018-22914-5, published online 16 March 2018

The original version of this Article contained an error in the title of the paper, where the word “availability” was incorrectly given as “available”. This has now been corrected in the PDF and HTML versions of the Article, and in the accompanying supplementary material.

