# Supplementary material for: Analyses of HIV-1 integrase sequences prior to South African national HIV-treatment program and availability of integrase inhibitors in Cape Town, South Africa
Source: Sci Rep. 2018 Mar 16;8:4709. doi: 10.1038/s41598-018-22914-5 (PMC5856838; doi:10.1038/s41598-018-22914-5)
Supplement: Supplementary file 1 — Supplementary Table 1 [file 41598_2018_22914_MOESM1_ESM.docx]

**Research Article**

**Title: Analyses of HIV-1 integrase sequences prior to South African national HIV-treatment program and availability of integrase inhibitors in Cape Town, South Africa**

Short title: **INSTI DRM from South Africa**

**Dominik Brado**^1#^**, Adetayo Emmanuel Obasa**^2,3#*^**, George Mondinde Ikomey**^4^**, Ruben Cloete**^5^**, Kamalendra Singh^3,6,7^, Susan Engelbrecht**^2^**, Ujjwal Neogi**^3^**, Graeme Brendon Jacobs**^2^

^1^Division of Virology, Institute for Virology and Immunobiology, Faculty of Medicine, University of Wuerzburg, 97080, Wuerzburg Germany

^2^Division of Medical Virology, Department of Pathology, Faculty of Medicine and Health Sciences, Stellenbosch University, Tygerberg, 7505, Cape Town, South Africa

^3^Division of Clinical Microbiology, Department of Laboratory Medicine, Karolinska Institute, University of Stockholm, Sweden

^4^CSCCD, Faculty of Medicain and Biomedical Sciences, University of Yaoundé I, Cameroon

^5^South African Medical Research Council Bioinformatics Unit, South African National Bioinformatics Institute, University of the Western Cape.

^6^Department of Molecular Microbiology and Immunology and ^7^Christopher Bond Life Sciences Center, University of Missouri, Columbia MO 65211, USA

^#^Both authors contributed equally to the work

*Corresponding author: Mr. Adetayo Emmanuel Obasa (obasa@sun.ac.za)

**Supplementary Table 1: Demographical and clinical data of patients**

| Isolate | Race / Gender | Orientation | Symptoms | Subtype (IN gene) |
| --- | --- | --- | --- | --- |
| TV0116 | CF | Hetero | Asymptomatic | C |
| TV0118 | CF | Hetero | Asymptomatic | C |
| TV0122 | CM | MTCT | Asymptomatic | B |
| TV0124 | BF | Hetero | Shingles, Miositis | C |
| TV0126 | BM | Hetero | Asymstomatic | C |
| TV0127 | BM | Hetero | symp to stage II | C |
| TV0128 | BF | Hetero | TB | C |
| TV0132 | BF | Hetero | Weight loss, Oral candidiasis, Pneumonia | C |
| TV0133 | BM | Hetero | TB | C |
| TV0135 | CF | Hetero | Asymstomatic | C |
| TV0137 | BF | Hetero | DVT, Ca CervixIIIB, prev Radiotherapy | C |
| TV0139 | BF | Hetero | Asymstomatic | C |
| TV0141 | BM | Hetero | Asymstomatic | C |
| TV0142 | BF | Hetero | Asymstomatic |  |
| TV0143 | BM | Hetero | TB treatment completed | C |
| TV0144 | BF | Hetero | Asymstomatic | C |
| TV0145 | BF | Hetero | STD | C |
| TV0147 | BF | Hetero | Asymstomatic | C |
| TV0148 | BM | Hetero | Current pleural effusion | C |
| TV0149 | CF | MTCT | Pneumonia x2 | C |
| TV0152 | BM | Hetero | Asymptomatic | C |
| TV0153 | BM | Hetero | Asymptomatic | B |
| TV0155 | BM | Hetero | Asymptomatic | C |
| TV0157 | BM | Hetero | TB | C |
| TV0160 | CM | Hetero | Asymptomatic | C |
| TV0161 | BF |  |  | C |
| TV0163 | CF | Hetero | Sin I with MPV | C |
| TV0164 | CF |  | Asymptomatic | C |
| TV0165 | CF | Hetero | PTB | C |
| TV0166 | BF |  |  | C |
| TV0168 | CF | Hetero | Candida | C |
| TV0173 | BM | Hetero | Symptomatic of TB | C |
| TV0175 | CF | Hetero | Seborrhea | C |
| TV0177 | BF | Hetero | Anal Warts | C |
| TV0180 | BF | MTCT | Septicemia, PID, Lymphoma, | C |
| TV0181 | BF | Hetero | Asymptomatic | C |
| TV0182 | BF | Hetero | Asymptomatic | C |
| TV0183 | BF | Hetero | Asymptomatic | C |
| TV0184 | BF | Hetero | Asymptomatic | C |
| TV0185 | IF |  |  | C |
| TV0191 | BF |  |  | C |
| TV0193 | BF |  |  | C |
| TV0198 | BF |  |  | C |
| TV0201 | BF |  |  | C |
| TV0202 | BF |  |  | C |
| TV0345 | BF | Hetero | Asymptomatic | C |
| TV0346 | BF | Hetero | Asymptomatic | C |
| TV0347 | CF | Hetero | Asymptomatic | C |
| TV0348 | BM | Hetero | Oral thrush, Prurigo, Weight Loss, Diarrhoea | C |
| TV0349 | BM | Hetero | Asymptomatic | C |
| TV0350 | BM | Hetero | Weight Loss, Oral thrush | C |
| TV0353 | CF | Hetero |  | C |
| TV0356 | CF | Hetero | Asymptomatic | B |
| TV0364 | BF | Hetero | Focal neurologic deficits | C |
| TV0365 | CM | Hetero | Opthamic Zoster, Candidiasis, Severe Psoriases | C |
| TV0366 | CF | Hetero | Thrombocytopenia | C |
| TV0367 | CF | Hetero | Asymptomatic | C |
| TV0370 | BM | Hetero | Asymptomatic | C |
| TV0371 | BF | Hetero | Asymptomatic | C |
| TV0372 | BF | Hetero | TB | C |
| TV0373 | BM | Hetero | Prurigo, Weight loss, Lymphadenopathy | C |
| TV0375 | BF | Hetero | TB<1year, Genital Warts, Candidiasis | C |
| TV0376 | BF | Hetero | Asymptomatic | C |
| TV0377 | BM | Hetero | Asymptomatic | C |
| TV0388 | CF |  |  | C |
| TV0398 | BM | Hetero |  | C |
| TV0404 | CF | Hetero | Asymptomatic | B |
| TV0405 | BF | Hetero | Asymptomatic | C |
| TV0406 | BF | Hetero |  | C |
| TV0407 | BF | Hetero | Prurigo, Lymphadenopathy | C |
| TV0412 | BM | Hetero | Chronic staph. Aur. skin sepsis | A |
| TV0413 | CF | Hetero | Asymptomatic | C |
| TV0417 | CF | Hetero |  | C |
| TV0418 | BF | Hetero |  | C |
| TV0420 | CF |  |  | B |
| TV0424 | BM | MTCT | PTB | C |
| TV0425 | BF | Hetero | Asymptomatic | C |
| TV0426 | BF | Hetero | Prurigo | C |
| TV0427 | BF | Hetero | Asymptomatic | C |
| TV0431 | WM | Hetero | Asymptomatic | B |
| TV0432 | BF |  | Asymptomatic | C |
| TV0433 | CF | Hetero | Asymptomatic | C |
| TV0434 | BF | Hetero | TB | C |
| TV0435 | BF | Hetero | Asymptomatic | C |
| TV0436 | BM | Hetero | Asymptomatic | C |
| TV0437 | BM | Hetero | Asymptomatic | C |
| TV0438 | BM | Hetero | TB | C |
| TV0442 | WF | Hetero |  | C |
| TV0445 | Mix | Hetero | Asymptomatic | C |
| TV0446 | CF | Hetero | Asymptomatic | C |
| TV0456 | CF | Hetero | Asymptomatic | C |

**BF**: Black Female, **BM**: Black Male, **CF**: Coloured Female, **CM**: Coloured Male, **WF**: White Female, **WM**: White Male, **PTB**: Pulmonary Tuberculosis, **DVT**: Deep Vein Thrombosis: **STD**: Sexually Transmitted Diseases
